# Stress Is a Latent Construct: Exploring the Differential Experience of Stress Among Black Older Adults

**DOI:** 10.1093/geroni/igab046.718

**Published:** 2021-12-17

**Authors:** Lauren Brown, Catherine Garcia, Alexis Reeves, John Pamplin, Uchechi Mitchell

**Affiliations:** 1 San Diego State University School of Public Health, San Diego State University School of Public Health, California, United States; 2 University of Nebraska - Lincoln, Lincoln, Nebraska, United States; 3 University of Michigan, Ann Arbor, Michigan, United States; 4 NYU Center for Urban Science and Progress, Brooklyn, New York, United States; 5 University of Illinois Chicago, School of Public Health, Chicago, Illinois, United States

## Abstract

While evidence highlights the detrimental health consequences of stress exposure for Black Americans, the impact of stress exposure on health varies by the stressor, individual appraisal and coping mechanisms examined. In this study, we aim to explore the differential effects of chronic stress exposure by means of latent class analysis on mental and physical health. Data come from 800 Black older adults ages 52+ from the 2006 Health and Retirement Study. A set of items that include stress exposure, appraisal and coping were used to assess chronic stress burden on anxiety, depressive symptoms and chronic conditions to identify stress and health clusters. Analysis revealed four subgroups, each demonstrated a typological response pattern with the most pronounced health consequences for high stress exposure, appraisal and few or no coping mechanisms. Results show an alternative approach to examining the stress-health link by using a combined person- and variable-centered approach.

